# Exploring the Phenomenon of Loneliness among Older Adult Informal Caregivers: An Integrative Review

**DOI:** 10.1093/geroni/igaf122.3586

**Published:** 2025-12-31

**Authors:** Alyssa Abbott, Jane Flanagan, Karen Lyons

**Affiliations:** Boston College, Chestnut Hill, Massachusetts, United States; Boston College, Chestnut Hill, Massachusetts, United States; Boston College, Chestnut Hill, Massachusetts, United States

## Abstract

As caregivers age alongside their care recipients, they face unique health challenges, including loneliness. While aging and informal caregiving have been established as risk factors for loneliness, less is known about its manifestation among older adult caregivers. This integrative review, conducted in accordance with the Preferred Reporting Items for Systematic Reviews and Meta Analysis (PRISMA) guidelines, aimed to synthesize current research on loneliness in older adult informal caregivers. Seventeen studies met the inclusion criteria. All but one study were descriptive in nature. One cross-sectional study used a mixed methods approach to explore the impact of an online communication strategy on loneliness. Of the remaining studies, nine were qualitative, four were secondary analysis, one employed a survey design. Per inclusion criteria, all participants were aged 65 or older. Across studies, caregiver characteristics were often underreported; however, when available, most caregivers were female (e.g., 72%) and caring for a spouse. Only one study focused exclusively on male caregivers, and few reported race, ethnicity, or other sociodemographic variables. Findings highlighted that caregiving transitions, particularly involuntary separation due to long-term care placement and the end-of-life period, were associated with heightened loneliness. Notably, loneliness was rarely assessed or addressed by healthcare providers. Loneliness is a salient concern among older adult informal caregivers, especially during caregiving transitions. Recognizing the intersecting stressors faced by this population is essential for informing the design of interventions and guiding policies to better support caregiver well-being.

